# Electroencephalography resting‐state networks in people with Stroke

**DOI:** 10.1002/brb3.2097

**Published:** 2021-03-23

**Authors:** Dylan B. Snyder, Brian D. Schmit, Allison S. Hyngstrom, Scott A. Beardsley

**Affiliations:** ^1^ Biomedical Engineering Marquette University and Medical College of Wisconsin Milwaukee WI USA; ^2^ Department of Physical Therapy Marquette University Milwaukee WI USA

**Keywords:** asymmetry, connectivity, EEG, orthogonalization, power, stroke

## Abstract

**Introduction:**

The purpose of this study was to characterize resting‐state cortical networks in chronic stroke survivors using electroencephalography (EEG).

**Methods:**

Electroencephalography data were collected from 14 chronic stroke and 11 neurologically intact participants while they were in a relaxed, resting state. EEG power was normalized to reduce bias and used as an indicator of network activity. Correlations of orthogonalized EEG activity were used as a measure of functional connectivity between cortical regions.

**Results:**

We found reduced cortical activity and connectivity in the alpha (*p* < .05; *p* = .05) and beta (*p* < .05; *p* = .03) bands after stroke while connectivity in the gamma (*p* = .031) band increased. Asymmetries, driven by a reduction in the lesioned hemisphere, were also noted in cortical activity (*p* = .001) after stroke.

**Conclusion:**

These findings suggest that stroke lesions cause a network alteration to more local (higher frequency), asymmetric networks. Understanding changes in cortical networks after stroke could be combined with controllability models to identify (and target) alternate brain network states that reduce functional impairment.

## Graphical abstract

The purpose of this study was to characterize the reorganization of resting‐state cortical networks after stroke using electroencephalography (EEG). We found reduced cortical activity and connectivity in the alpha and beta bands after stroke while connectivity in the gamma band increased. These findings suggest that stroke lesions cause a network reorganization to more local (higher frequency), asymmetric networks.

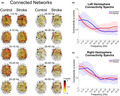

## 1 INTRODUCTION

The purpose of this study was to characterize resting‐state cortical networks in chronic stroke survivors using electroencephalography (EEG). Functional magnetic resonance imaging (fMRI), magnetoencephalography (MEG), and EEG all reveal regions of the brain that have common activation patterns which are thought to be representative of functionally connected networks (Aoki et al., 2015; Biswal et al., 1995; Brookes et al., 2011; Rosazza & Minati, 2011; Wirsich et al., 2020). Some of the more common cortical networks include the default mode, sensorimotor, executive control, visual, lateralized frontoparietal, auditory, and temporoparietal networks (Aoki et al., 2015; Biswal et al., 1995; Brookes et al., 2011; Rosazza & Minati, 2011). The brain utilizes these cortical networks in different ways including memory consolidation, cognition, vision, and movement (Bressler, 1995; Corbetta, 1998; Mazoyer et al., 2001; Sukerkar, 2010). An improved understanding of the changes in these networks after stroke could provide insight into the mechanisms underlying functional loss and recovery.

FMRI studies of brain networks following stroke indicate altered cortical patterns throughout the brain including areas within the default mode, sensorimotor, executive control, visual, and lateralized frontoparietal networks (Balaev et al., 2018; Zhao et al., 2018). Using fMRI, Tuladhar and colleagues found reduced functional connectivity after stroke within and between the medial temporal lobe, posterior cingulate, and medial prefrontal cortex; areas associated with the default mode network (Tuladhar et al., 2013). FMRI studies of sensorimotor network activity during motor tasks have shown increased activity in both hemispheres excluding the lesioned region (Carey et al., 2002; Grefkes et al., 2008; Mintzopoulos et al., 2009; Rossini et al., 1998; Ward et al., 2003). Others have shown a corresponding decrease in functional connectivity within and between hemispheres during motor tasks (Grefkes et al., 2008; Mintzopoulos et al., 2009). While fMRI offers excellent spatial resolution (~1 mm) of the cortex, it lacks temporal resolution (~1 s) which prevents the study of underlying brain processes that act at the millisecond time scale (Koenig et al., 2005; Lopes da Silva, 2013). EEG and MEG, with their better temporal resolution (~1 ms), have been employed to overcome this issue.

EEG/MEG studies of brain networks after stroke indicate frequency‐specific changes. Sensorimotor task‐based studies in people with stroke show impairment‐specific changes in alpha and beta band activity, with a decrease in activity near the lesion and an increase in cortical asymmetry (Platz et al., 2000; Rossiter et al., 2014; Stępień et al., 2011; Strens et al., 2004). When examining EEG functional connectivity during a visually guided grip task, Bönstrup and colleagues found alpha band connectivity increased within the lesioned motor networks of stroke patients (Bönstrup et al., 2018). Despite their usefulness, task‐based studies tend to be limited to stroke participants who can perform the tasks and can result in mirror movements that confound the results of the study (Calautti et al., 2007; Dong et al., 2006; Ward et al., 2007; Weiller et al., 1993; Wittenberg et al., 2000).

Resting‐state paradigms, where participants remain still and relaxed, have the advantage of including participants of all functional abilities and are easier and quicker to administer than task‐based paradigms. After stroke, resting‐state EEG shows increased bilateral power in the delta and theta bands as well as increased power asymmetries between hemispheres (Assenza et al., 2013; Köpruner & Pfurtscheller, 1984; Wang et al., 2012). EEG resting‐state studies have also reported decreased connectivity in the alpha and beta bands within the lesioned area (Dubovik et al., 2012, 2013; Wu et al., 2015).

The alteration of resting‐state EEG frequency characteristics after stroke may be representative of underlying structural changes. Brain networks with a large neuronal population or spatial extent oscillate at lower frequencies (Bullock et al., 1995; Eckhorn, 1994; Kopell et al., 2000; von Stein & Sarnthein, 2000). This observation led Nunez to develop a theoretical framework for the inverse relationship between frequency of activity and spatial scale of a network (Nunez, 2000). Further, local sensory integration invokes gamma band activity, multisensory integration produces upper alpha and lower beta band activity, while long‐range interactions involve theta and alpha band activity (von Stein & Sarnthein, 2000). Gamma band synchronization decreases with distance, with lower frequency oscillations associated with longer‐range interactions (Bullock et al., 1995; Eckhorn, 1994; Kopell et al., 2000). These cortical network frequency dependencies arise from the physical architecture of the networks, speed of communication due to axon conduction/synaptic delays, and the number of synapses involved in the network path (Nunez, 1995; von Stein et al., 2000). Thus, it is important to consider spectral information in the interpretation of EEG activity and connectivity data.

While resting‐state EEG networks in people with stroke indicate frequency specific changes in cortical activity and connectivity, there are elements of the analysis that confound the identification of the networks and hamper interpretation of the resulting data. The analysis of resting‐state EEG is influenced by electrode impedance, neuronal density under each electrode and volume conduction. The power of resting‐state EEG is affected by electrode impedance such that electrodes with lower impedance display higher power. In addition, larger synchronous neuronal populations beneath an electrode produce greater signal power, which may influence interpretation of the signal size, especially in people with loss of brain tissue after stroke. EEG estimates of functional connectivity are also affected by volume conduction. Volume conduction results in significant connectivity between EEG electrodes that can extend over distances of up to 8 cm (Nunez et al., 1997), even if the cortical regions immediately below the electrodes are not functionally connected. Imaginary coherence (Nolte et al., 2004), orthogonalization techniques (Brookes et al., 2012; Hipp et al., 2012), and other phase metrics that exclude zero lag connectivity (Nolte et al., 2008) can be used to mitigate this issue.

In this study, we set out to quantify the changes in resting‐state cortical network power and connectivity in people with chronic stroke. We collected EEG data while participants were in a relaxed, resting state. EEG power was normalized to reduce bias and used as an indicator of network activity. Correlations of orthogonalized EEG activity were used to measure functional connectivity between cortical areas. We hypothesized that cortical networks are more asymmetric after stroke and that there is a shift in the frequency due to changes in cortical communication after stroke. Specifically, we expected cortical networks to have a higher reliance on local network activity with less efficient pathways connecting local regions, resulting in a shift to higher frequency.

## 2 MATERIALS AND METHODS

### 2.1 Participant population

A sample of 14 chronic stroke participants and 11 age‐matched neurologically intact controls participated in this study. Stroke participants (8 male, aged 36–79 years) were required to be at least 1‐year poststroke. Prior to the study, participants completed a questionnaire and were asked to self‐report known sensory and motor deficits, including visual deficits due to either physical deficits (e.g., homonymous hemianopsia) or to inattention disorders. Exclusion criteria included the diagnosis of any other neurological disorder or recent treatment that interfered with neuromuscular function, such as botulinum toxin injection. The impairment level of stroke participants was assessed using the upper extremity Fugl‐Meyer Assessment (FMA) which consists of a motor portion (maximum score 66) and sensory/proprioception portion (maximum score 12; Fugl‐Meyer et al., 1975) and the Semmes‐Weinstein monofilament test (Semmes et al., 1960). The monofilament test was performed at seven locations on the palmar surface of the paretic hand and averaged (distal phalanx of the small finger, index finger and thumb; proximal phalanx of the small and index finger; thenar, and hypothenar). Control participants (7 male, aged 34–77 years) reported no history of stroke or any other neuromuscular pathology. Detailed demographic data for all participants are shown in Table 1. All participants gave written informed consent, and all procedures were approved by the Marquette University Institutional Review Board in accordance with the Declaration of Helsinki.

**TABLE 1:** Demographic and clinical data for stroke (S) and control (C) participants.

Subject Identifier	Sex	Age (year)	Time after Stroke (year)	Fugl‐Meyer (Motor:66)	Fugl‐Meyer (Sensory:12)	Monofilament (g,sensation)	
S1	F	60	23	63	12	0.08	(‐)
S2	F	79	7	62	11	0.15	(‐)
S3	F	67	30	29	12	0.05	(N)
S4	M	57	2	28	6	134.29	(‐‐‐)
S5	M	64	16	61	8	0.19	(‐)
S6	F	66	26	51	4	60.00	(‐‐‐)
S7	M	61	11	31	12	0.35	(‐)
S8	F	65	13	38	8	94.29	(‐‐‐)
S9	M	64	14	34	12	50.28	(‐‐‐)
S10	M	59	14	23	8	60.00	(‐‐‐)
S11	M	73	7	21	8	100.00	(‐‐‐)
S12	M	36	8	21	8	71.43	(‐‐‐)
S13	F	71	4	30	12	0.01	(N)
S14	M	55	15	27	12	37.43	(‐‐‐)
C1	F	68	‐	‐	‐	‐	
C2	M	64	‐	‐	‐	‐	
C3	M	61	‐	‐	‐	‐	
C4	M	51	‐	‐	‐	‐	
C5	F	77	‐	‐	‐	‐	
C6	F	57	‐	‐	‐	‐	
C7	M	67	‐	‐	‐	‐	
C8	M	65	‐	‐	‐	‐	
C9	F	63	‐	‐	‐	‐	
C10	M	34	‐	‐	‐	‐	
C11	M	64	‐	‐	‐	‐	

Note

“Fugl‐Meyer” indicates the Fugl‐Meyer upper extremity score (Motor: maximum of 66; Sensory: maximum of 12). Monofilament values indicate the average force in grams (g) across the seven hand locations tested with the degree of sensation (N: normal (*g* < 0.07), “‐”: diminished light tough (0.07 > g < 0.4), “‐‐”: diminished protective sensation (0.4 > g < 2.0), “‐‐‐”: loss of protective sensation (2.0 > g < 180.0), “‐‐‐‐”: deep pressure sensation only (g > 180)). (F: female; M: male; ND: non‐dominant)

### 2.2 Experimental protocol

During the study, participants were seated in a chair and asked to remain as still as possible, keep their eyes closed, refrain from making any eye movements, and clear their mind. EEG data were collected for approximately 3 min in this relaxed, resting state.

### 2.3 Physiological measurements

A 64‐channel active electrode actiCAP (Brain Products GmbH) system was used to record EEG data. EEG electrodes were arranged in the conventional 10–20 system with the reference at FCz and the ground at AFz. The EEG cap was placed on the participant's head such that the Cz electrode was in line with the preauricular points in the frontal plane and with the nasion and inion points in the sagittal plane. SuperVisc gel (Brain Products GmbH) was applied between the scalp and electrodes to lower the electrode impedances below 10 kOhms prior to data collection. EEG data were amplified, sampled at 1 kHz, filtered from 0.1 to 200 Hz and notch filtered at 60 Hz using a Synamps^2^ amplifier system (Neuroscan), and recorded using the Neuroscan software, Scan 4.5. The data that support the findings of this study are available from the corresponding author upon reasonable request.

### 2.4 Data analysis

Electroencephalography data were postprocessed and analyzed using the EEGLAB toolbox (version v13.4.4b; Delorme & Makeig, 2004) for storing and configuring the data, FieldTrip (version 2016‐01‐03; Oostenveld et al., 2011) for removing bad epochs and electrodes, Brainstorm (version 3.4; Tadel et al., 2011) for source localization, Network Based Statistic Toolbox (version 1.2; Zalesky et al., 2010) for statistically comparing network connectivity, BrainNet Viewer (version 1.62; Xia et al., 2013) for visualizing network connectivity and custom MATLAB scripts (version 2014a, MathWorks, Natick, Massachusetts). All EEG data were bandpass filtered (1‐50 Hz) using a fourth order zero‐phase Butterworth filter. Trial epochs of the EEG data were then extracted by creating 2 s, consecutive, nonoverlapping windows starting at the beginning of the file and continuing until a complete 2 s window could not be formed. This process resulted in approximately 90 epochs per participant. EEG epochs were then zero‐meaned, and bad channels and epochs removed manually using FieldTrip's visual inspection code (channel/epoch removed if variance/kurtosis >2 standard deviations from the mean, “ft_rejectvisual,” average number channels/epochs removed, 0.8/10.4, per subject). If a channel was rejected from the EEG data, its value was replaced with interpolated data from the surrounding electrode channels. Stroke participant EEG data were flipped so that the hemisphere associated with the lesion was always represented on the left. EEG data were then separated into signal and artifactual components using an adaptive mixture independent component analysis (AMICA; Palmer et al., 2008), with 64 independent temporal components. Signal artifacts, including electromyography and movement artifacts, were identified by distinct artifactual characteristics (Delorme et al., 2012; Makeig et al., 2004; Mognon et al., 2011; Puce & Hämäläinen, 2017) and removed from the EEG data (average number of artifact components removed, 6.6; minimum number: 3; maximum number: 15). The remaining components were then transformed back to the EEG channel space. Finally, EEG data were re‐referenced to a common average reference for all data analyses. The re‐reference technique reintroduced the FCz electrode to the data set. For the following analyses, EEG data were separated into ten non‐overlapping 5 Hz frequency bands ranging from 1 to 50 Hz (first band only ranged from 1–5 Hz due to the 1 Hz high pass filter applied during preprocessing) to determine whether frequency shifts occurred in the stroke group relative to the controls.

A power spectrum analysis was performed at the electrode level to examine the spatial characteristics of resting‐state EEG power across frequency bands. The power spectrum at every electrode was calculated using Welch's method with epochs as the measure of consistency (Welch, 1967). The frequency bands were then extracted from the power spectrum and normalized at each electrode using Equation 1,

(1) $$\text{NP}\;=\;100\;\times \;\frac{\sum F}{\sum \text{Total}},$$

where NP represents the normalized power, ∑F represents the sum of power within a frequency band, and ∑Total represents the sum of power across the frequency spectrum (1–50 Hz). By normalizing power in this fashion, we could determine whether the cortical area's function (distribution of power across the spectrum) was changing while removing any dependence on electrode impedance or neuronal population size. To characterize any effects that stroke lesions may have on the spatial distribution of frequency, the control and stroke groups were compared at every electrode within each frequency band using a two‐sample *t* test with a false discovery rate (FDR) of *α* = 0.05 for multiple comparisons correction. To facilitate interpretation of normalized power, average absolute power within frequency bands was computed and plotted for each electrode to determine whether normalized power differences between controls and stroke survivors were due to true absolute power changes within frequency bands or if a normalization bias was driving the normalized power differences. For instance, a loss of absolute power in one frequency band could result in normalized power increases in other frequency bands, even though they are not changed on an absolute level. Similar to normalized power, absolute power for control and stroke groups was compared at every electrode within each frequency band using a two‐sample *t* test with a false discovery rate (FDR) of *α* = 0.05 for multiple comparisons correction.

To determine whether frequency bands displayed power differences between hemispheres, an electrode directional asymmetry metric was computed between analogous electrodes in the two hemispheres using Equation 2,

(2) $$\text{EDA}\;=\;100\;\times \;\frac{1}{n}\sum \frac{{\text{NP}}_{L}‐{\text{NP}}_{R}}{\left|{\text{NP}}_{L}\right|+\left|{\text{NP}}_{R}\right|},$$

where EDA is the whole head electrode directional asymmetry, NP*_L_* is the normalized power of the homologous electrode in the left hemisphere, NP*_R_* is the normalized power of the homologous electrode in the right hemisphere, and *n* is the total number of electrode pairs. Electrodes along the midline were ignored for calculation of the EDA. This metric resembles the brain symmetry index created by van Putten and colleagues (van Putten et al., 2004); however, it has been modified to allow for the directionality of any asymmetries to be identified similar to what was done by Saes and colleagues (Saes et al., 2019).

Volume source localization of EEG data was performed to enable volumetric connectivity analyses. Distributed current dipole volumes were computed in Brainstorm using the default MNI/ICBM152 anatomical brain template with the cerebellum included (Tadel et al., 2011). The standard actiCAP electrode locations were fit to the scalp surface so that the Cz electrode location was at the vertex as described in the physiological measurements section. A boundary element model (BEM) was used to estimate the forward model (OpenMEEG; Gramfort et al., 2010; Kybic et al., 2005) with volumetric vertices (5 × 5 × 5 mm) placed on a regular grid spanning the entire brain. A depth‐weighted minimum L2 norm estimator of current density (Hämäläinen & Ilmoniemi, 1994) was used to estimate the inverse model where each vertex consisted of three orthogonal dipoles (representing the *x*, *y*, and *z* directions). The three‐dimensional dipole activity for every vertex was subsequently processed using a principal component analysis (PCA) to obtain a single activity time course that best represented the volumetric source.

**FIGURE 1:**
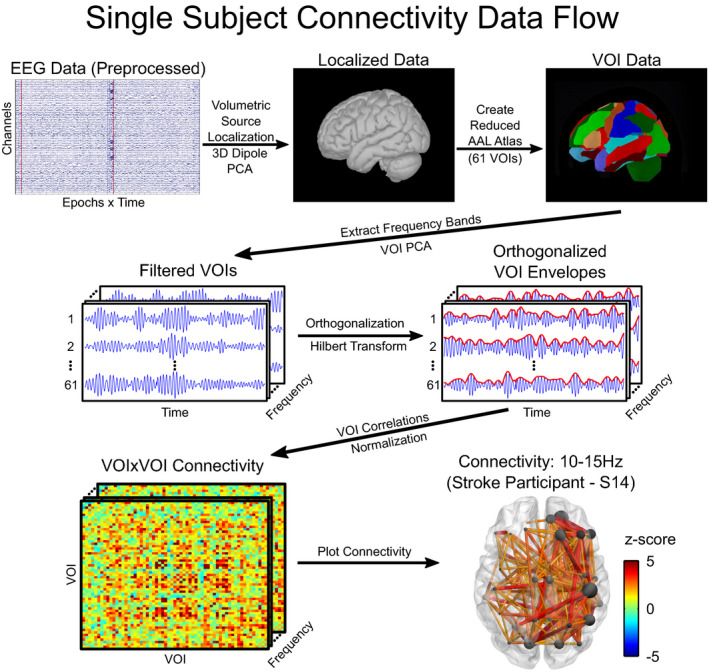
Diagram of connectivity workflow and the 10–15 Hz frequency band connectivity for a single stroke participant (S14) thresholded at a *z*‐score of ±2. After preprocessing, EEG data were projected into a volumetric source space where a PCA was applied to each three‐dimensional dipole to extract the time course that best represented the dipole's activity. The brain was then segmented into 61 VOIs based on a reduced AAL atlas. All VOIs were filtered voxel‐wise to extract the frequency bands of interest, and PCA was applied to reduce the dipole activity within the VOIs to a single time course. For each frequency band, VOI time series were orthogonalized after which the envelope of the VOI activity was obtained via the Hilbert transform. Correlations between the envelopes of VOI activity were performed within frequency bands to characterize connectivity between VOI’s. For the representative stroke subject shown (S14), the hemisphere associated with the stroke lesion is displayed on the left. Stronger connections between nodes are represented by larger z‐scores and line widths. Node size indicates the number of connections a node makes with other nodes

Following the projection of EEG data into volumetric source space, the functional connectivity between all brain regions was calculated within the defined frequency bands, Figure 1. First, the source localized data were bandpass filtered using a zero‐phase fourth order Butterworth filter to extract the different frequency bands; the resulting data were then concatenated across epochs within frequency bands. A reduced version (described below) of the Automated Anatomical Labeling (AAL) atlas (Tzourio‐Mazoyer et al., 2002) from the micron software package (https://people.cas.sc.edu/rorden/mricron/index.html) was used to define volumes of interests (VOI) for input into the connectivity analysis. Reductions to the original AAL atlas VOIs were necessary because we intended to orthogonalize the VOI time courses to reduce the effect of volume conduction on connectivity analyses. Orthogonalization by way of a symmetric multivariate correction (Colclough et al., 2015) is dependent on the rank of the data (which was limited to 61 due to one participant only having a maximum of 61 valid electrodes after preprocessing). Therefore, we reduced the original 116 AAL atlas VOIs to 61 VOIs, Figure 1. The 12 subcortical structures (left and right) were left unaltered, while the 9 cerebellar VOIs within each hemisphere and 8 vermis VOIs were merged, respectively. The 34 cortical VOIs in the left hemisphere were reduced to 23 iteratively by finding the smallest VOI and merging it with the nearest VOI based on VOI centroid locations. The homologous VOIs merged in the left hemisphere were then merged in the right hemisphere to maintain a symmetrical VOI distribution.

Once the reduced AAL atlas was defined, PCA was performed across the voxel time courses within each VOI with the largest component of the PCA retained, resulting in a single activity time course that best represented each VOI. All VOI time courses were then orthogonalized using a symmetric multivariate correction with twenty iterations (Colclough et al., 2015) to reduce the EEG volume conduction artifact and spatial leakage that results from source localization estimates. Power envelopes of the orthogonalized VOI time courses were then calculated by taking the absolute value of their Hilbert transform, a similar approach for calculating activity envelopes has been done in previous studies (Brookes et al., 2011; Hipp et al., 2012). VOI power envelopes were then correlated within frequency bands resulting in a connectivity matrix of correlation coefficients that was 61 (number of VOIs) by 61 (number of VOIs) by 10 (number of frequency bands) for each participant. Connectivity correlation coefficients were Fisher z‐transformed to normalize the sample distribution for statistical analysis. An additional normalization was performed across frequency bands to account for the inverse relationship between correlation and frequency band for a fixed time window. For the bias normalization, we performed a Monte Carlo simulation using 1,000 iterations on the pipeline described above by randomizing the VOI time series phase information while retaining the magnitude information. This resulted in a random “noise” correlation distribution for each participant, frequency band and VOI‐to‐VOI interaction. The true Fisher *z*‐transformed connectivity data were then bias corrected by subtracting the mean and dividing by the standard deviation of the random “noise” distributions converting the true connectivity data to a *z*‐score relative to the null distribution.

To determine whether frequency bands displayed connectivity differences between hemispheres, a connectivity directional asymmetry metric was computed between analogous connections in the two hemispheres using Equation 3,

(3) $$\text{CDA}\;=\;100\;\times \;\frac{1}{n}\sum \frac{C_{L}‐C_{R}}{\left|C_{L}\right|+\left|C_{R}\right|},$$

where CDA represents the whole brain connectivity directional asymmetry metric, *C_L_* represents the connectivity of the homologous connections in the left hemisphere, *C_R_* represents the connectivity of the homologous connections in the right hemisphere, and *n* represents the total number of homologous connection pairs. Connectivity between homologous regions was ignored.

To visualize frequency dependent shifts in the stroke population relative to the control population, connectivity spectra (connectivity vs. frequency) were plotted for connections within the left (lesioned) and right (non‐lesioned) hemispheres. To quantify deviations in the shape of the connectivity spectrum from the control group, the connectivity spectrum of each participant (control and stroke) was correlated with the average connectivity spectrum from the control population. Finally, connectivity spectrum correlation values were Fisher *z*‐transformed to normalize the sample distribution for statistical testing.

The Network Based Statistic toolbox (Zalesky et al., 2010) was used at the group level to identify significantly connected networks in the control and stroke groups and networks that were significantly different between sample groups. The Network Based Statistic, a graph analogue of cluster‐based statistical methods, used permutation testing to control the family‐wise error rate (*p* <.05) associated with multiple comparisons tests based on the extent (number of connections in a network) of the network above a predefined (defined by the user) threshold. For our analysis, we tested networks that were either positively or negatively correlated between VOI’s. We modified the Network Based Statistic code (added the capability to perform one‐sample *t* tests) to compute the network statistics within the control (threshold: *t*‐value = 3.169) and stroke (threshold: *t*‐value = 3.012) groups using a one‐sample *t* test (thresholds for both groups were equivalent to a two‐tailed one‐sample *t* test *p*‐value of .01). Significant differences between the networks of control and stroke groups (threshold: *t*‐value = 2.5) were identified using a two‐sample *t* test applied to the Network Based Statistic (the threshold for differences between groups was equivalent to a two‐tailed two‐sample *t* test *p* value of .02).

### 2.5 Statistical analysis

Changes in electrode and connectivity directional asymmetry were characterized across subjects using a two‐way mixed ANOVA with frequency as the within‐subject factor and group as the between‐subject factor in the analysis. One‐way ANOVAs and *t* tests were applied post hoc to characterize specific interaction effects identified in the two‐way ANOVAs. Changes in the connectivity spectra correlation between groups were characterized by using a two‐sample *t* test. If Mauchly's Test of Sphericity indicated that the assumption of sphericity was violated, a Greenhouse–Geisser correction was used for the ANOVA results. The Holm–Sidak method for correcting for multiple comparisons was used at each level (between multiple ANOVAs and *t* tests) of the analysis except for the pairwise comparisons where the Tukey post hoc test was applied. Raw *p*‐values were reported and stated as significant if they survived the correction for multiple comparisons. A non‐parametric bootstrap approach similar to the Zhou and Wong method (Zhou & Wong, 2011) with 10,000 iterations was used to generate the statistical distributions for the Tukey post hoc test. Statistical tests were performed with a Type I error rate of *α* = 0.05. If significant differences were identified between the control and stroke groups for the electrode normalized power distributions, electrode directional asymmetry, connectivity directional asymmetry, connectivity spectra, or connectivity networks analysis, a simple linear regression analysis was performed with the variable of interest and the upper extremity motor FMA for the stroke participants. Plots were displayed whether the regression analysis was significant (*p* < .05). Data are reported as mean ± *SD* unless stated otherwise.

## 3 RESULTS

### 3.1 Normalized power: Control population

**FIGURE 2:**
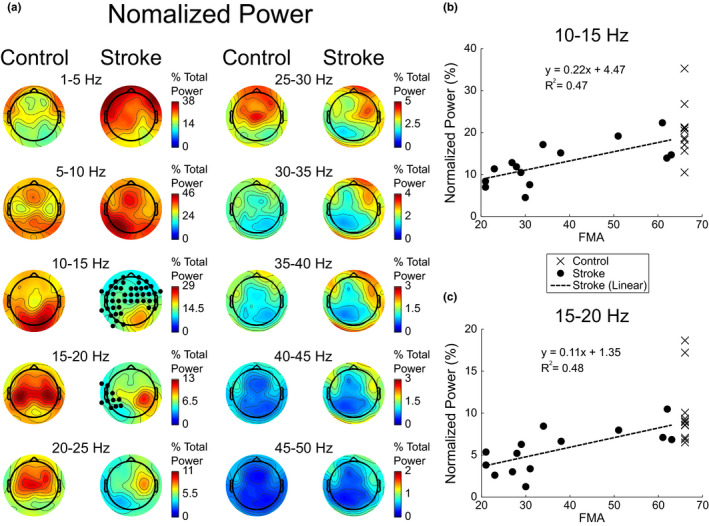
Electrode power and linear regression analysis during resting state. The hemisphere associated with the stroke lesion is displayed on the left. (a) Topographic maps of the normalized electrode power averaged across participants are shown for each group and frequency band of interest. Black dots indicate electrodes whose power was significantly different between the control and stroke groups, using an FDR correction of *α* = 0.05 (please refer to Figure S1 for an alternative visualization of significant power differences). Values are interpolated between electrodes for visualization purposes. (b and c) Linear regression of the stroke group normalized power averaged across significantly different electrodes and upper extremity motor FMA scores for the 10–15 and 15–20 Hz frequency bands, respectively. Normalized power for each frequency band was plotted against a perfect upper extremity motor FMA of 66 for controls

Electrode level normalized power was examined to identify the spatial distribution of power across electrodes for each frequency band of interest and to determine whether the power distribution was different between control and stroke groups (Figure 2a). In the controls, the lower half of the frequencies examined (1‐25 Hz) accounted for ~85% of the total power while the upper half of the frequencies (25–50 Hz) contributed ~15%. The regions that contributed the most power in the 1–5 Hz frequency band were located above the bilateral frontal cortices while for the 5–10 Hz band, the power was largest above the medial frontal cortices and the medial/lateral parietal cortices. There was a posterior to anterior shift in the regions that contributed the most power for the frequency bands ranging from 10 to 50 Hz, with regions located above the bilateral visual cortices for the 10–15 Hz band, two nodes located above the bilateral parietal/sensory/motor cortices for the 15–20 Hz band, a region located above the bilateral motor/premotor cortices for the 20–25 Hz band, a region located above the bilateral premotor/frontal cortices for the 25–30 Hz band, and regions located over the bilateral frontal cortices for the remaining frequency bands (30–50 Hz).

### 3.2 Normalized power differences

The normalized power of the stroke group was similar to the controls, particularly for the distribution of power across frequency bands. However, within frequency bands the normalized power from 1–10 and 30–50 Hz was larger in the stroke group while the normalized power from 10 to 25 Hz was smaller in the stroke group when compared to the control group (Figure 2a). In general, absolute power within frequency bands displayed differences between the stroke and control groups similar to the normalized power. Absolute power from 1–10 and 30–50 Hz was larger in the stroke group while the absolute power from 10 to 25 Hz was smaller in the stroke group when compared to the control group (Figure S2).

To assess significant changes in spatial power distribution, normalized power was compared between the control and stroke groups at every electrode within each frequency band. Even though there were changes in power across all frequency bands examined, only the 10–15 and 15–20 Hz frequency bands resulted in significant differences (*p* <.05) following FDR correction. In the stroke group, the 10–15 Hz band contained significantly less power across the entire brain except above the right (non‐lesioned) visual cortex (averaged across significantly different electrodes; control group: 20.18 ± 6.42% total power; stroke group: 12.6 ± 4.94% total power) while the 15–20 Hz band contained significantly less power across electrodes located over the left (lesioned) sensory/parietal cortices (Figure 2a) compared with controls (averaged across significantly different electrodes; control group: 10.08 ± 4.05% total power; stroke group: 5.6 ± 2.57% total power). Across significantly different electrodes, average normalized power was significantly related to motor function (upper extremity motor FMA) in both the 10–15 and 15–20 Hz bands (*R* ^2^ = 0.47, *p* =.007 and *R* ^2^ = 0.48, *p* =.006, respectively; Figure 2b,c). When assessing significant changes in spatial power distribution of absolute power between the control and stroke groups at every electrode within each frequency band, no frequency bands revealed electrodes with significant differences following FDR correction (Figure S2).

### 3.3 Normalized power asymmetry

**FIGURE 3:**
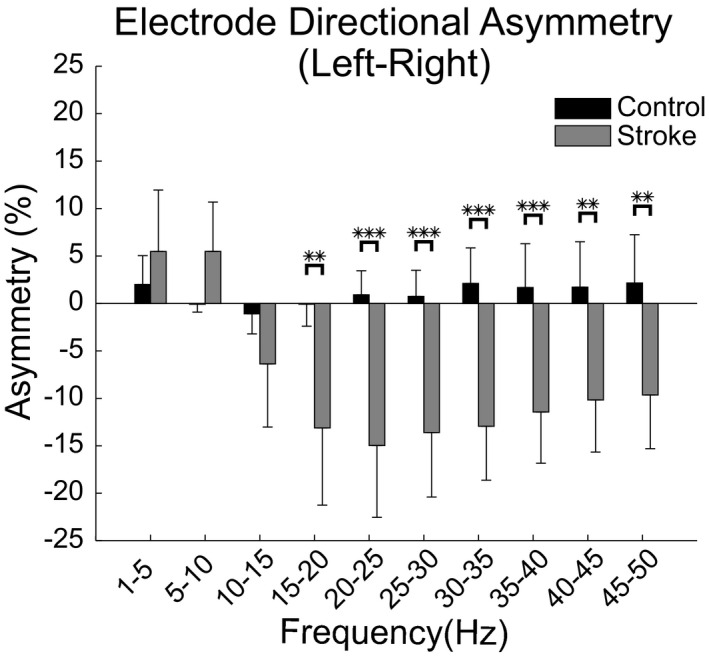
Directional asymmetry in electrode power during resting‐state EEG. The directional asymmetry in electrode power averaged across participants is shown for each group and frequency band of interest. Positive asymmetry values indicate that the frequency band had larger normalized power in the left (lesioned) hemisphere while negative asymmetry values indicate the right (non‐lesioned) hemisphere had larger normalized power. Error bars denote the 95% confidence interval about the mean. Significant differences determined via post hoc analysis (Tukey test) are indicated by stars (*p <.05, **p <.01, and ***p <.001)

In addition to the differences in normalized power, the power topographies for the stroke group were more asymmetric when compared to those of the control group (Figure 2a). In the stroke group, power in the 1–10 Hz frequency bands showed larger power in the left (lesioned) hemisphere while the 10–50 Hz frequency bands exhibited larger power in the right (non‐lesioned) hemisphere. The two‐way ANOVA for differences in electrode directional asymmetry indicated a main effect of frequency (*F*(2.70,62.04) = 6.47, *p* =.001) and group (*F*(1,23) = 16.21, *p* =.001) with an interaction effect between frequency and group (*F*(2.70,62.04) = 6.88, *p* =.001). The post hoc two‐sample *t* tests for group differences within frequencies indicated that the frequency bands ranging from 15 to 50 Hz (averaged across 15–50 Hz frequency bands; control group: 1.31 ± 4.73%; stroke group: −12.27 ± 9.84%) were significantly (*t*(23) > 2.99, *p *< .007) more asymmetric in the stroke group with the power being larger in the right (non‐lesioned) hemisphere, Figure 3. The 5–10 Hz (control group: −0.84 ± 1.24%; stroke group: 5.48 ± 9.02%) frequency band approached significance (*t*(23) = 2.02, *p* =.055) with more power found in the left (lesioned) hemisphere while the frequency bands from 1–5 Hz (control group: 2.0 ± 4.53%; stroke group: 5.48 ± 11.22%) and 10–15 Hz (control group: −1.09 ± 3.16%; stroke group: −6.37 ± 11.52%) were not significantly (*t*(23) < 1.47, *p *> .15) different between the control and stroke groups. The post hoc one‐way ANOVAs for frequency showed significant differences between frequencies for the stroke group (*F*(2.40,31.16) = 9.83, *p* =.0003) but not the control group (*F*(2.32,23.21) = 0.76, *p* =.498). The post hoc analysis (Tukey test) of frequency within the stroke group indicated that the electrode directional asymmetry was significantly different between the frequency bands in the 1–10 Hz range and all other frequency bands (*q*(117) > 4.91, *p *< .025) while no other frequency bands showed significant differences (*q*(117) < 3.56, *p *> .27). Linear regression analysis of electrode directional asymmetry (for significantly different frequency bands) and function (upper extremity motor FMA) indicated that directional asymmetry was not a good predictor of motor function (*R* ^2^ < 0.2, *p *> .11).

### 3.4 Functional connectivity: Networks

**FIGURE 4:**
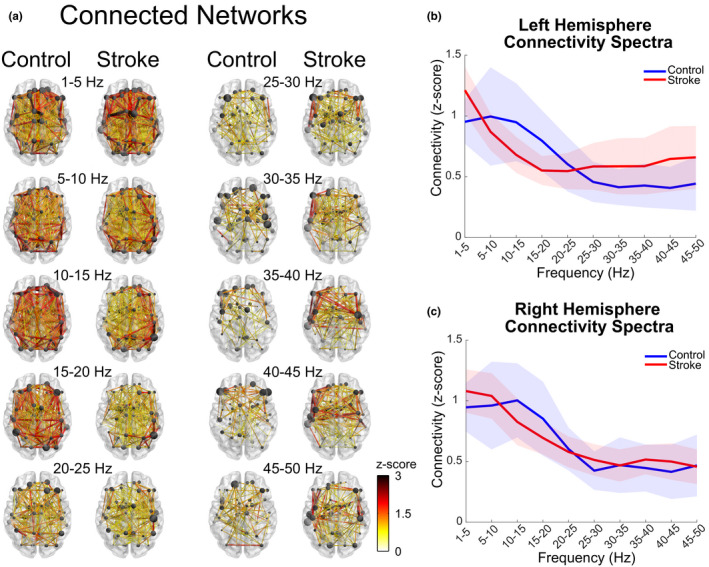
Functional connectivity networks and connectivity spectra during resting state. The hemisphere associated with the stroke lesion is displayed on the left. (a) Networks deemed significantly connected within control (*p *< .0008) and stroke (*p *< .0002) groups are shown for each frequency band of interest. Stronger connections between nodes are represented by larger *z*‐scores (color) and line widths. Node size indicates the degree of connectivity (number of connections a node makes with other nodes) and is normalized by the maximum degree within each frequency band of interest for each group. Please refer to Figure S3 for an alternative visualization of functional connectivity networks. (b and c) Left (lesioned) and right (non‐lesioned) hemisphere connectivity spectra, respectively. Average connectivity of all connections (not just the significantly connected connections) within the for each frequency band of interest. Shaded areas indicate the 95% confidence interval about the mean

Networks identified via functional connectivity analysis were examined to identify the spatial extent of connectivity for each frequency band of interest and to determine whether the connectivity spectra were different between stroke and control groups. No networks defined by negative correlations were found in the control (*p *> .9568) or stroke groups (*p *> .9999). All networks described below resulted from positive correlations between VOIs in the control (*p *< .0008) and stroke (*p *< .0002) groups. In the control group, connectivity was stronger (i.e., higher correlations) and more extensive at lower frequencies (1–20 Hz), which peaked in the 5–15 Hz frequency range. In contrast, higher frequencies (25–50 Hz) exhibited fewer connections that tended to be located in the anterior half of the brain (Figure 4a). Connectivity spectra for the control group in the left and right hemispheres (Figure 4b,c), mirrored the frequency dependences shown in the network plots (Figure 4a). The high connectivity in the lower frequencies (1–20 Hz) sloped downward until it reached a plateau in the higher frequencies (25–50 Hz).

The stroke group showed similarities to the control network patterns; however, there were also notable differences. Lower frequencies (1–20 Hz) had more extensive functional connectivity throughout the brain while higher frequencies (25–50 Hz) had fewer connections that tended to be located in the anterior half of the brain (Figure 4a). However, for the stroke group, connectivity in the 5–20 Hz frequency bands tended to be more asymmetric with lower connection strength occurring in the left (lesioned) hemisphere. Conversely, stroke networks in the 25–50 Hz frequency bands contained more (and larger) connections. The two‐way ANOVA for differences in connectivity directional asymmetry indicated that there was a main effect of frequency (*F*(4.58,105.24) = 2.89, *p* =.003), no effect of group (*F*(1,23) = 0.75, *p* =.109), and a trend toward significance in the interaction effect between frequency and group (*F*(2.26,105.24) = 4.58, *p* =.06). The post hoc analysis (Tukey test) of frequency indicated that the connectivity directional asymmetry was significantly different between the 10–15 Hz band and the 1–5/40–45 Hz bands (*q*(216) > 4.73, *p *< .03), trended toward a significance difference between the 1–5 Hz and 15–20 Hz bands (*q*(216) = 4.27, *p* =.08) and showed no differences for the remaining frequencies (*q*(216) < 4.07, *p *> .119). While there was no significant interaction effect between frequency and group for the connectivity asymmetry analysis (*p* =.06), the stroke group data did seem to drive the results found in the main effect of frequency. In general, the stroke group had larger connectivity directional asymmetry in all bands with the 5–25 Hz bands (averaged across 5–25 Hz frequency bands; control group: 0.04 ± 2.69%; stroke group: −3.29 ± 5.52%) displaying larger connectivity in the right (non‐lesioned) hemisphere and 1–5/25–50 Hz bands (averaged across 1–5/25–50 Hz frequency bands; control group: 0.65 ± 2.07%; stroke group: 2.28 ± 3.18%) displaying larger connectivity in the left (lesioned) hemisphere.

When comparing the connectivity spectra between groups (Figure 4b,c), the left (lesioned) hemisphere (control group: 1.37 ± 0.49 Fisher *Z*; stroke group: 0.52 ± 1.02 Fisher *Z*) was significantly different (*t*(23) = 2.55, *p* =.018) while the right (non‐lesioned) hemisphere (control group: 1.28 ± 0.43 Fisher *Z*; stroke group: 0.92 ± 0.67 Fisher *Z*) showed no differences (*t*(23) = 1.08, *p* =.29) between stroke and controls. The only noticeable differences in the right (non‐lesioned) hemisphere connectivity spectrum of the stroke group were that it peaked in the 1–10 Hz range instead of the 5–15 Hz range while the connectivity in the 10–20 Hz frequency was slightly lower. On the contrary, the left (lesioned) hemisphere of the stroke group displayed a different spectrum entirely, peaking in the 1–5 Hz frequency band, sloping downward until the 15–20 Hz frequency band and gradually increasing throughout the 15–50 Hz frequencies. The stroke group's left (lesioned) hemisphere connectivity spectrum also showed a decrease in connectivity for the 5–25 Hz frequency bands and an increase in connectivity for the 25–50 Hz bands when compared to the control group. Linear regression analysis of the left connectivity spectrum and function (upper extremity motor FMA) showed a limited correspondence between measures (*R* ^2^ = 0.18, *p* =.13).

### 3.5 Functional connectivity: Different networks

**FIGURE 5:**
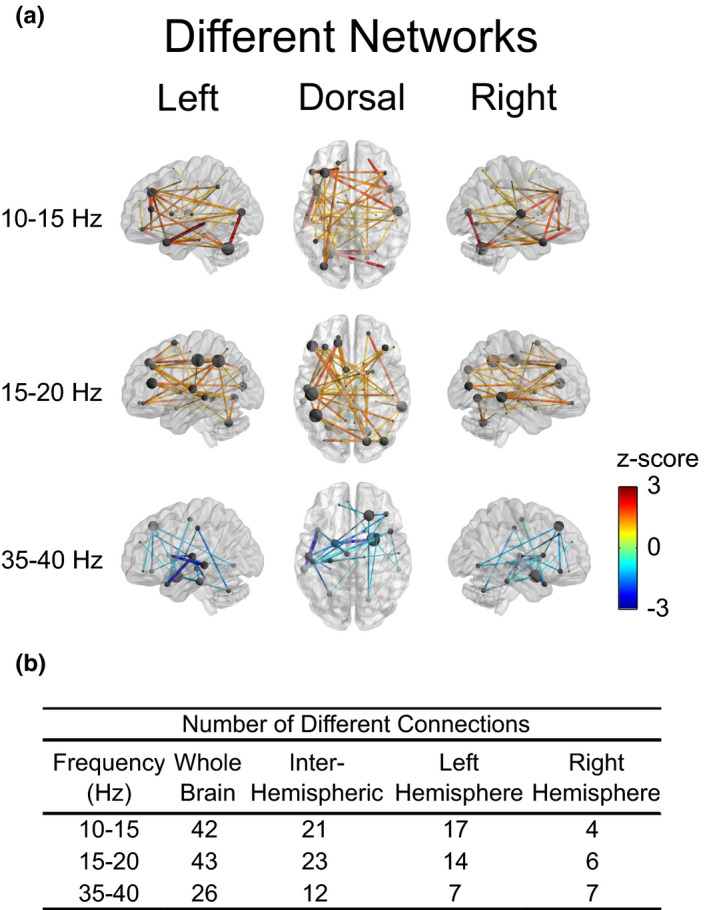
Resting‐state functional connections with statistically significant differences (*p* < .05) between control and stroke groups. The hemisphere associated with the stroke lesion is displayed on the left. (a) Networks with statistically significant differences across frequency bands. *Z*‐values correspond to the differences between the control and stroke groups with a positive or negative *z*‐value indicating stronger connections in the control or stroke group, respectively. Larger differences in connectivity are also denoted by larger line widths between nodes. Node size indicates the degree (number of connections a node makes with other nodes) and is normalized by the maximum degree within each frequency band of interest. Please refer to Figure S4 for an alternative visualization of functional connectivity networks. (b) Comparison of the numbers of inter‐ and intrahemispheric connections within networks that were significantly different between groups

To better visualize and quantify the changes between resting‐state connectivity in the control and stroke groups, we identified networks that were significantly different between groups within each frequency band of interest (Figure 5a). Note that here we define “network” as a group of connections within a frequency band. Networks with significantly larger connectivity in the control group occurred in the 10–15 Hz (*p* =.05; average network connectivity; control group: 0.90 ± 0.48 *z*‐score; stroke group: 0.59 ± 0.24 *z*‐score) and 15–20 Hz (*p* =.03; average network connectivity; control group: 0.77 ± 0.44 *z*‐score; stroke group: 0.50 ± 0.21 *z*‐score) frequency bands. In the stroke group, one network showed significantly larger connectivity in the 35–40 Hz (*p* = .031; average network connectivity; control group: 0.44 ± 0.36 *z*‐score; stroke group: 0.59 ± 0.54 *z*‐score) frequency band while another network in the 30–35 Hz frequency band approached significance (*p* =.066). No other frequency bands contained significantly different networks (*p *> .18). The 10–15 and 15–20 Hz networks with significantly larger connectivity in the control group included connections throughout the brain; however, there were more connections in the left (lesioned) hemisphere compared with the right (non‐lesioned) hemisphere (Figure 5a,b). The 10–15 Hz network included nodes with high degree (degree > 4) located in the left inferior frontal, middle frontal, middle/inferior occipital, middle/superior temporal pole and right middle/superior temporal pole, and heschl/rolandic operculum/superior temporal regions with the highest degree occurring in the left cerebellum (degree = 8). The 15–20 Hz network included nodes with high degree (degree > 4) located in the left inferior frontal, superior frontal, heschl/rolandic operculum/superior temporal, cerebellum and right lingual, middle temporal, inferior/middle occipital, cuneus/superior occipital regions with the highest degree occurring in the left angular/inferior parietal and left postcentral/supramarginal regions (degree = 6). The 35–40 Hz network present in the stroke group was localized toward the anterior portion of the brain with an equal number of connections in the left (lesioned) and right (non‐lesioned) hemispheres (Figure 5a,b) The 35–40 Hz network included nodes with high degree in the right superior frontal gyrus (degree = 4) and right amygdala (degree = 5). In all three networks with between‐group differences, approximately 50% of the connections occurred between hemispheres (Figure 5a,b). For the networks that were different between groups, no significant relationship was found between average connectivity and motor function (upper extremity motor FMA, *R* ^2^ < 0.05, *p *> .43).

## 4 DISCUSSION

### 4.1 Main Results

In this study, we set out to identify the changes in resting‐state cortical signal power and connectivity in people with chronic stroke. We hypothesized that cortical activity (power) and connectivity are more asymmetric after stroke and that there is a shift in the frequency of cortical communication. The results demonstrated that cortical activity patterns after stroke display asymmetric patterns and that shifts in the frequency of communication occur. Specifically, during resting state, stroke cortical network activity (EEG normalized power) in the upper frequency ranges (15–50 Hz) became more asymmetric (electrode directional asymmetry) with less activity occurring in the lesioned hemisphere (Figures 2 and 3). The cortical network activity identified in stroke was lower in the alpha and lower beta bands (10–20 Hz), suggesting a disruption of normal cortical activity (Figure 2). The level of network connectivity in the stroke group (correlation of orthogonalized EEG band envelope activity) was reduced in the alpha and beta bands (10–20 Hz) and increased in the gamma band (35–40 Hz) when compared to controls (Figures 4 and 5). These differences in connectivity were driven by changes occurring within the lesioned hemisphere (Figures 4 and 5). The shift from typical alpha/beta band connectivity to increased gamma band connectivity suggests an alteration to more local cortical networks after stroke. The presence of decreased cortical activity, increased cortical activity asymmetries, and shifts in cortical connectivity indicate the disruption of typical cortical networks and an alteration to more local networks after stroke.

### 4.2 Patterns of resting‐state power

In controls, lower frequencies contributed the most to the total power with different frequency bands exhibiting different spatial topographies (Figures 2 and 3). Areas with larger normalized power were located above the bilateral frontal cortices for the 1–5 Hz band, above the medial frontal cortices and the medial/lateral parietal cortices for the 5–10 Hz band while there was a bilateral posterior to anterior shift for the frequency bands ranging from 10 to 50 Hz. Previous EEG studies examining resting state have shown similar topography patterns for the delta (1–4 Hz), theta (4–8 Hz), alpha (8–12 Hz), and gamma (>30 Hz) bands with lower frequencies containing higher power; however, spatial topographies for the beta (13–30 Hz) band in previous studies were found to be focused above the bilateral occipital regions (Barry et al., 2007; Chen et al., 2008; Qin et al., 2010). The differences in beta band spatial topography between our results and previous literature was due to our choice to normalize the power. We chose to normalize power to remove the influence of electrode impedance and underlying population size on absolute power measures. Normalized power provides insight into how cortical areas are functioning (relative weighting of frequency band powers) and may result in spatial shifts when compared to absolute power measures which indicate the cortical areas with the largest source (raw power). When examining the absolute power of our data (Figure S2), the spatial distributions of all frequency bands were consistent with previous literature.

The different spatial patterns of EEG normalized power suggest that specific cortical regions are associated with specific frequency bands. Theta/alpha (5–10 Hz) band power was shown to have larger normalized power above the medial frontal cortices and the medial/lateral parietal cortices suggesting a relationship to the default mode network, Figure 2. Specifically, the regions with the highest power in the theta/alpha frequency band resided over the medial prefrontal gyrus, anterior cingulate, posterior cingulate, and the angular gyri, which are all nodes associated with the default mode network (Damoiseaux et al., 2006; Muldoon et al., 2016; Raichle et al., 2001). EEG theta power has been shown to negatively correlate with the fMRI blood oxygen level dependent (BOLD) signal within the default mode network (Scheeringa et al., 2008). The alpha/beta (10–15 Hz) band displayed the largest normalized power above the bilateral visual cortices hinting at an association with the visual network, Figure 2. Alpha band activity is known to relate to visual stimulation/processing and shows decreased power during increased levels of visual stimuli (Barry et al., 2007; Chen et al., 2008; Gale et al., 1969, 1971). Further, the EEG alpha band power has been shown to be correlated with the fMRI BOLD signal in visual occipital areas during resting state (Goldman et al., 2002; Scheeringa et al., 2012). The largest amount of beta (15–30 Hz) band power was localized over the bilateral parietal, sensory, motor, and premotor cortices which links the beta band to the sensorimotor network. These regions have all been shown to modulate beta band activity during the control of movement in EEG event‐related desynchronization studies (Pfurtscheller & Lopes da Silva, 1999; Pfurtscheller et al., 1997, 1999). Although the current results support previous literature linking EEG resting‐state power to well defined cortical networks, EEG theta, alpha, and beta frequency bands should not be interpreted as being associated with only one cortical network or process. When examining resting state under either eyes open or eyes closed conditions, all frequencies ranging from delta to gamma show decreased power during the eyes open condition, possibly linking these bands to the arousal state of the cortex (Barry et al., 2007; Chen et al., 2008). In addition, resting‐state research involving fMRI, EEG, and MEG has also indicated that multiple frequency bands are associated with the default mode, sensorimotor, executive control, visual, lateralized frontoparietal, auditory, and temporoparietal networks (Aoki et al., 2015; Brookes et al., 2011; Mantini et al., 2007).

### 4.3 Power changes after stroke

The spatial topographies of normalized power observed in the stroke group differed substantially from controls. The stroke group had significantly lower power in the alpha/beta (10–15 Hz) band across the entire brain except for the right non‐lesioned visual cortex, Figure 2. Interestingly, the changes in the alpha/beta band power after stroke were not localized to areas above the visual cortices but were found globally across the scalp. While the alpha band has been related to the visual network (Barry et al., 2007; Chen et al., 2008; Gale et al., 1969, 1971; Goldman et al., 2002; Scheeringa et al., 2012), stroke participants in our study did not report any stroke‐related visual deficits such as loss of visual field or visuospatial neglect. However, the reported widespread decreases in the alpha/beta band could be related to changes in visual processing. Visual processing changes after stroke have been identified in studies examining visual memory performance and visual attention (Lange et al., 2000; Mazer et al., 2001). Alternatively, the widespread decreases in stroke alpha/beta (10–15 Hz) band power could be representative of an altered baseline arousal or activity state of the cortex after stroke. Alpha band activity has been shown to reduce power when the brain is more aroused in an eyes open versus eyes closed state (Barry et al., 2007; Chen et al., 2008). Further, alpha band resting‐state power shares an inverse relationship to cortical activity (Goldman et al., 2002; Scheeringa et al., 2012), suggesting the resting brain might be in a state of higher arousal or activity after stroke.

Analysis of the stroke group's spatial normalized power distribution within the beta (15–20 Hz) band revealed significantly lower power localized to areas over the lesioned hemisphere's sensory/parietal cortex (Figure 2) indicative of dysfunction in the sensorimotor network. This result supports previous literature showing altered beta band activity above the lesioned motor cortex after stroke (Platz et al., 2000; Rossiter et al., 2014). Both the 10–15 and 15–20 Hz levels of power in the stroke group correlated with impairment, supporting past research indicating that functional ability can be predicted from resting‐state information (Assenza et al., 2013; Kawano et al., 2017; Wu et al., 2015). While no electrodes showed significant differences within the delta (1–5 Hz) and theta (5–10 Hz) frequency bands in our study, both frequency bands did show increases in normalized power relative to the controls supporting previous findings from Assenza indicating that delta and theta band powers are increased after stroke (Assenza et al., 2013).

When examining absolute power, it was found that absolute power differences between the stroke and control groups mimicked the trends seen in normalized power. This indicates that the observed differences in normalized power between the control and stroke groups were likely due to true absolute power changes within frequency bands as opposed to a bias arising from normalization. While we focused our analysis on normalized power in the present study, analyzing absolute power should not be neglected because it offers valuable insight into the interpretation of normalized power results.

The stroke group had greater electrode directional asymmetry in the upper (15–50 Hz) frequency bands with less power in the lesioned hemisphere, Figure 3. Previous studies have shown that stroke patients tend to have more asymmetric EEG power distributions and can be classified into either a stroke or control group based on their level of asymmetry (Köpruner & Pfurtscheller, 1984). Underlying networks, including the default mode network, are also more asymmetric after stroke (Tuladhar et al., 2013). The increased asymmetry is likely due to both a loss of neural substrate in the lesioned cortex as well as a shift in functional activity to the contralesional cortex due to cortical plasticity associated with recovery (James et al., 2009; Johansen‐Berg et al., 2002; Liepert et al., 2000).

### 4.4 Patterns of resting‐state connectivity

In controls, theta/alpha/beta (5–15 Hz) frequency band connections were the most numerous, had the largest values of connectivity, and were symmetrically distributed throughout the cortex, suggesting they may be the dominant frequencies for cortical communication during resting state (Figure 4). Similar connectivity profiles with peaks in connectivity in the alpha and beta bands have been observed in resting‐state MEG (Brookes et al., 2011; Hipp et al., 2012). The widespread distribution of connections found in the theta/alpha/beta frequency bands may be attributed to the fact that these frequencies are associated with multiple resting‐state cortical networks distributed throughout the brain (Aoki et al., 2015; Brookes et al., 2011; Mantini et al., 2007). Interestingly, networks were only revealed when examining positive connectivity correlations as opposed to negative connectivity correlations. This indicates, at least under the constraints of our connectivity pipeline, that the brain's resting‐state connectivity between regions may be dominated by excitatory versus inhibitory interactions.

### 4.5 Connectivity changes after stroke

In comparison with the connectivity patterns seen in controls, the stroke group's connectivity displayed lower, more asymmetric connectivity values in the theta/alpha/beta (5–15 Hz) frequency bands with larger connectivity values in the upper (25–50 Hz) frequencies (Figure 4). When testing for significant differences in connectivity between the control and stroke groups, we found networks with decreased connectivity in the alpha/beta (10–20 Hz) bands and increased connectivity in the gamma (35–40 Hz) band for the stroke group (Figure 5). Around half the connections of the significantly different networks in the alpha, beta, and gamma bands were identified to be interhemispheric connections, adding further evidence to notion that stroke disrupts inter‐hemispheric communication (Carter et al., 2009; Pellegrino et al., 2012). The decrease in connectivity observed in the alpha/beta band networks after stroke consisted of connections in both hemispheres, with most of the connections lateralized to the lesioned hemisphere, consistent with research showing deficits in functional connectivity throughout the brain but mainly in the lesioned hemisphere (Crofts & Higham, 2009; Crofts et al., 2011; De Vico Fallani et al., 2009; Tuladhar et al., 2013). Decreased connectivity of the alpha and beta bands in resting‐state paradigms has been reported using other EEG approaches (Dubovik et al., 2012, 2013; Wu et al., 2015). The decreased alpha (10–15 Hz) connectivity within a prefrontal‐cerebellar network in the stroke group is consistent with previous findings from our laboratory indicating decreased fMRI functional connectivity in a similar network after stroke (Kalinosky et al., 2017). The beta (15–20 Hz) band decreased connectivity consisted of prominent nodes in the lesioned hemisphere's sensory/parietal regions indicating it may be a marker of sensorimotor dysfunction that often occurs after stroke (Inman et al., 2012; Platz et al., 2000; Rossiter et al., 2014; Sharma et al., 2009; Wu et al., 2015). Although most connectivity research shows a reduction in functional connectivity after stroke, our finding of increased connectivity in the gamma (35–40 Hz) band supports EEG and modeling results showing stroke lesions can result in increased connectivity and may do so as a compensatory mechanism (Alstott et al., 2009; Bönstrup et al., 2018).

Interestingly, we found that connectivity patterns did not share a relationship with motor impairment when analyzed using our approach. This finding is at odds with observations that EEG resting‐state connectivity predicts functional and behavioral outcomes after stroke (Dubovik et al., 2012, 2013; Wu et al., 2015). In addition, connectivity directional asymmetry only indicated a trend toward asymmetry after stroke, likely due to large standard deviations. This suggests that connectivity patterns are complex, with likely increases and decreases in different frequencies and regions of the brain that challenge a direct functional interpretation.

### 4.6 Functional alteration after stroke

The stroke group's loss of cortical activity and connectivity in the alpha/beta bands along with their increase of cortical activity and connectivity in the gamma band suggests a disruption of typical cortical networks with an alteration to more local cortical networks after stroke (Figures 2, 4 and 5). The shift to more local (higher frequency networks) is most prevalent within the lesioned hemisphere with the largest changes observed in the connectivity spectrum (Figure 4b,c). Cortical networks with smaller neuronal populations/spatial extent oscillate at higher frequencies than networks with larger neuronal populations/spatial extent (Bullock et al., 1995; Eckhorn, 1994; Kopell et al., 2000; von Stein & Sarnthein, 2000). The frequency of network oscillations may not only depend on the distance or size of the two connected sites but also the number of synapses involved in an interaction (von Stein et al., 2000). After stroke, lesions to the cortex disconnect pathways linking disparate cortical regions resulting in smaller, isolated, more local (high frequency) networks. Our interpretation of high frequency activity representing local network activity and low frequency activity representing large scale network activity is supported by Zhu and colleagues who examined different frequency bands of resting‐state fMRI in the stroke population (Zhu et al., 2015). Zhu and colleagues discovered that differences in neural activity between the stroke and control groups were frequency dependent with slower oscillations identifying widespread cortical areas and faster oscillations identifying local areas (Zhu et al., 2015).

### 4.7 Study limitations

The current experimental design controlled for several confounding factors, such as the raw power bias in resting‐state EEG and volume conduction in EEG connectivity. However, other factors may have impacted the EEG power and connectivity, including brain signals associated with trunk stabilization, EEG contamination by muscle activity and removal of EEG signal in the signal processing pipeline. During the study, participants were seated in a chair but were not otherwise restrained. Although participants were monitored throughout the experimental sessions for trunk movement, with no movement noted, the control and stroke groups might have engaged stabilizing trunk muscles differently, eliciting group‐specific changes in cortical activity not specifically related to EEG resting state. Other potential confounding factors arose in the EEG data processing pipeline. It is possible that the AMICA algorithm did not fully separate signals and artifacts, resulting in the removal of some cortical signals and/or the inclusion of some artifactual components in the subsequent source imaging and analysis.

Another possible limitation centers around performing the connectivity analysis at the source level as opposed to the sensor level. When performing connectivity analysis at the source level, accuracy of results vary depending on the choice of anatomical template, electrical model, inverse method, and connectivity metric (Mahjoory et al., 2017). However, when estimating the forward model, we chose to use a MNI/ICBM152 anatomical brain template with the BEM, which has advantages over spherical‐shell models (Vatta et al., 2010). We estimated cortical sources using the weighted minimum norm estimate as opposed to beamforming methods, which has been shown to be more accurate for cortical patch sources (Hincapié et al., 2017). Lastly, source level connectivity was performed using orthogonalized amplitude correlations that show better test–retest reliability than other volume conduction independent connectivity measures such as imaginary coherence (Colclough et al., 2016). While performing connectivity analyses at the sensor level avoids these issues, sensor connectivity estimates have other complications. Coherence (connectivity) is dependent on the reference electrode or referencing scheme (common average, linked mastoids, etc.), (Essl & Rappelsberger, 1998; Nunez et al., 1999; Rappelsberger, 1989). The use of a single electrode as the reference can inflate or deflate coherence values depending on the level of activity at the reference electrode; with higher values at the reference electrode being detrimental to coherence (Zaveri et al., 2000). Rappelsberger (Rappelsberger, 1989) suggested using a reference averaging technique, such as linked earlobes, to better approximate a zero‐potential reference and mitigate this issue. While the common average reference provides an alternative averaging technique, the tendency for EEG signals to be synchronized over large areas of the scalp can result in a common average reference remaining high. While both sensor level and source level connectivity analyses have their idiosyncrasies, we opted to use the source level approach to obtain a better approximation of how cortical regions of the brain are connected.

### 4.8 Clinical applications

The results reported here indicate that cortical networks are disrupted after stroke and show an alteration to more asymmetric, local networks. In future studies, it would be interesting to examine how targeted changes in sensory feedback impact the asymmetry of local cortical networks and whether increased network symmetry correlates with motor recovery following stroke. Poststroke motor function has been associated with reductions in the integrity of somatosensory pathways (Campfens et al., 2015; Zandvliet, van Wegen, et al., 2020), and recent findings from Zandvliet and colleagues suggest that recovery of somatosensory integrity over time may be necessary for full motor recovery (Zandvliet, Kwakkel, et al., 2020). Additionally, brain controllability studies indicate that application of external stimuli (e.g., via the application of neurofeedback, external sensory stimuli, or electrical stimulation) may be able to shift brain networks into different functional states (Gu et al., 2015; Muldoon et al., 2016). The application of targeted external sensory stimuli could also act to alter the asymmetric, local networks seen in resting state. Understanding how external stimuli affect resting‐state networks could help explain functional improvements in spasticity, balance control, arm tracking, arm stabilization, and hand function reported during application of tendon vibration and electrical stimulation (Celnik et al., 2007; Conrad et al., 2015; Conrad et al., 2011a, 2011b; Dewald et al., 1995; Levin & Hui‐Chan, 1992; Priplata et al., 2006; Wu et al., 2006).

## 5 CONCLUSIONS

After stroke, EEG signals shifted from dominant alpha/beta (10–20 Hz) band networks toward higher frequency (35–40 Hz) gamma networks. Decreases in cortical activity (normalized power) were found globally for the alpha (10–15 Hz) band and locally above the lesioned hemisphere for the beta (15–20 Hz) band; both displayed a linear relationship with functional ability. Asymmetries in EEG power were also noted for the 15–50 Hz frequencies, with less power in the lesioned hemisphere. Connectivity results revealed networks within the alpha (10–15 Hz) and beta (15–20 Hz) bands that were lowered after stroke while one network in the gamma (35–40 Hz) band displayed increased connectivity after stroke. Stroke‐related changes in cortical activity and connectivity showed the largest effect in the lesioned hemisphere. These findings suggest that stroke lesions disrupt pathways causing network alteration to more local, asymmetric networks.

## CONFLICTS OF INTEREST

No conflicts of interest, financial or otherwise, are declared by the author(s).

## AUTHOR CONTRIBUTIONS

Dylan B. Snyder: Conceptualization, Methodology, Software, Validation, Formal Analysis, Investigation, Data Curation, Writing—Original Draft, Writing—Review and Editing, Visualization, Project Administration. Brian D. Schmit: Conceptualization, Methodology, Resources, Writing—Review and Editing, Supervision, Project Administration, Funding Acquisition. Allison S. Hyngstrom: Conceptualization, Methodology, Investigation, Resources, Writing—Review and Editing, Supervision, Project Administration. Scott A. Beardsley: Conceptualization, Methodology, Resources, Writing—Review and Editing, Supervision, Project Administration, Funding Acquisition.

### PEER REVIEW

The peer review history for this article is available at https://publons.com/publon/10.1002/brb3.2097.

## Supporting information

Supplementary Material

## Data Availability

The data that support the findings of this study are available from the corresponding author upon reasonable request.
