# Supplementary material for: Electroencephalography resting‐state networks in people with Stroke
Source: Brain Behav. 2021 Mar 23;11(5):e02097. doi: 10.1002/brb3.2097 (PMC8119848; doi:10.1002/brb3.2097)
Supplement: Supplementary file 1 — Supplementary Material [file BRB3-11-e02097-s001.docx]

**Title (160 char):**

Electroencephalography Resting State Networks in People with Stroke

**Authors:**

Dylan B. Snyder ^1^

Scott A. Beardsley ^1^

Allison S. Hyngstrom ^2^

Brian D. Schmit ^1^

**Department and Institution:**

^1^ Biomedical Engineering, Marquette University and Medical College of Wisconsin, Milwaukee, Wisconsin

^2^ Department of Physical Therapy, Marquette University, Milwaukee, Wisconsin

**Abbreviated Title (40 char):**

EEG Resting State Networks in Stroke

**Corresponding Author:**

Name: Scott A. Beardsley

Email: scott.beardsley@marquette.edu

Address: PO Box 1881, Milwaukee, WI 53201-1881

Phone: 414-288-4448

Supplementary Document


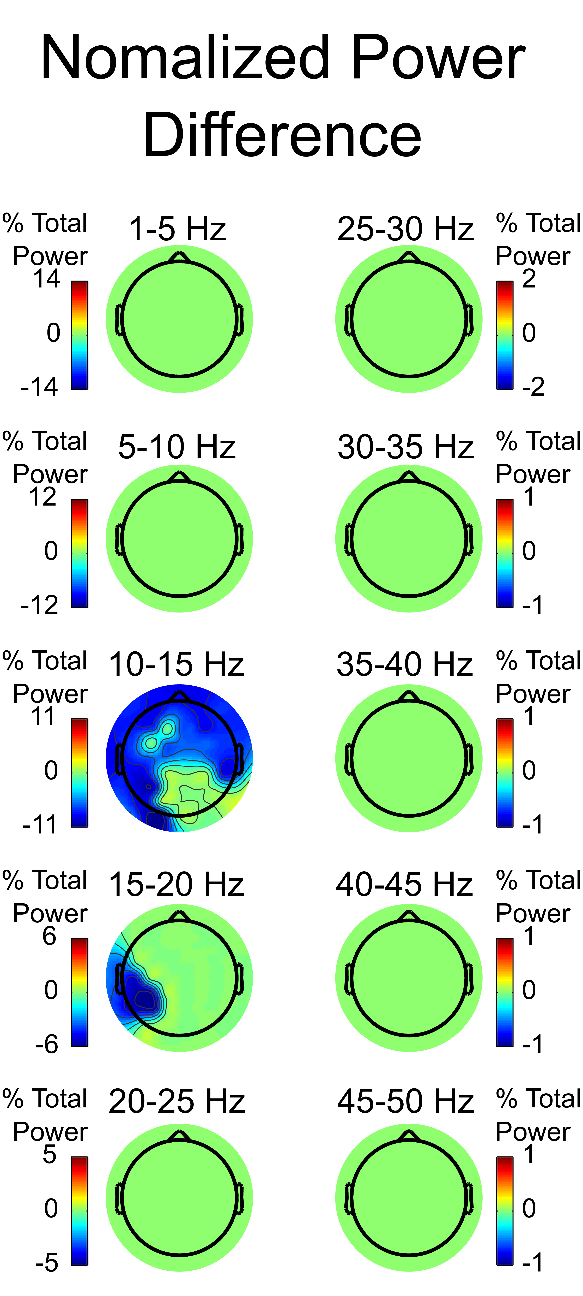


**Supplementary Figure 1:** Electrode normalized power differences during resting state. The hemisphere associated with the stroke lesion is displayed on the left. Topographic maps of the normalized electrode power differences (stroke group average – control group average) are shown for each frequency band of interest. Only electrodes whose power was significantly different between the control and stroke groups, using an FDR correction of α = 0.05, are displayed. Values are interpolated between electrodes for visualization purpose


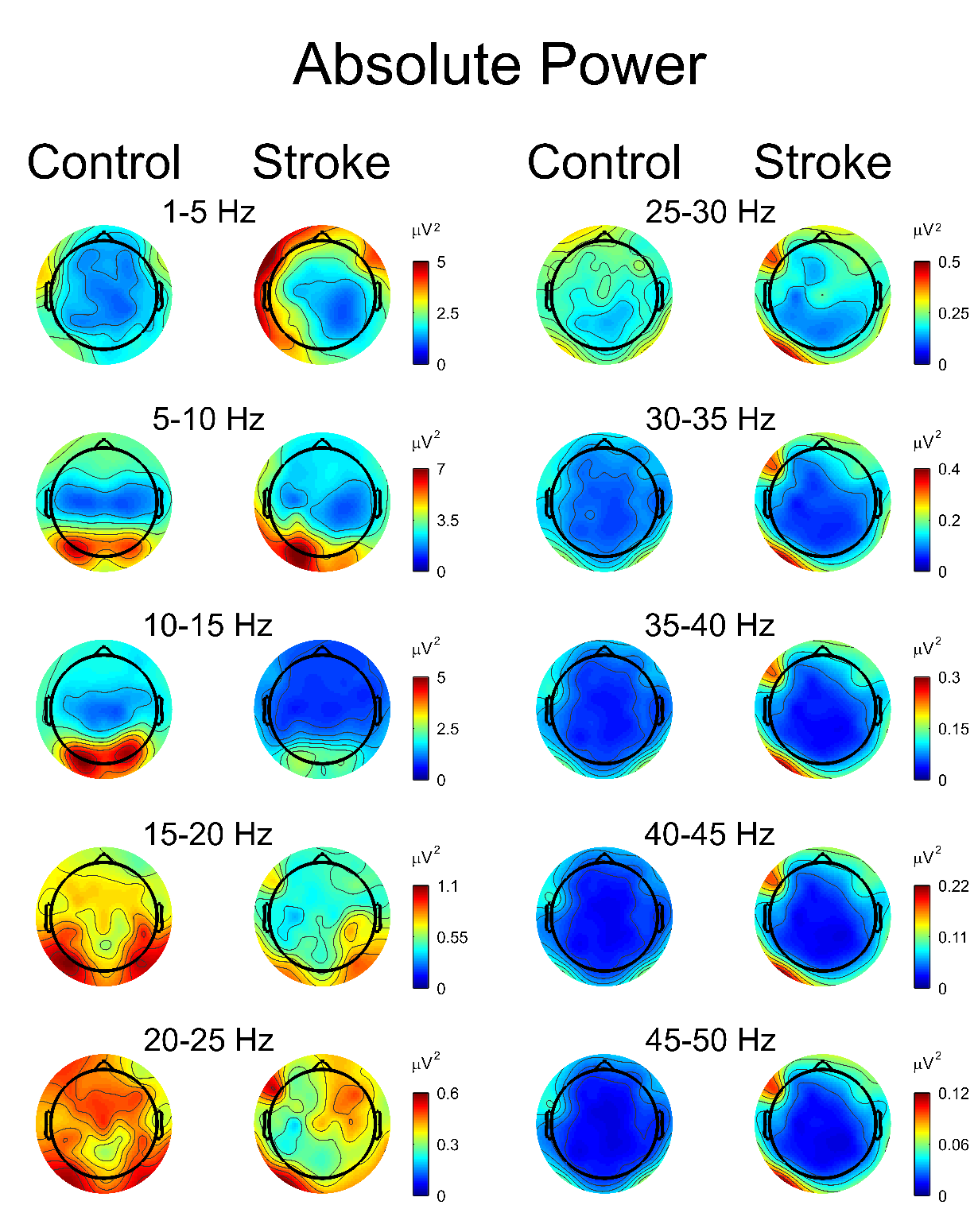


**Supplementary Figure 2:** Electrode absolute power during resting state. The hemisphere associated with the stroke lesion is displayed on the left. Topographic maps of the absolute electrode power averaged across participants are shown for each group and frequency band of interest. Values are interpolated between electrodes for visualization purposes.


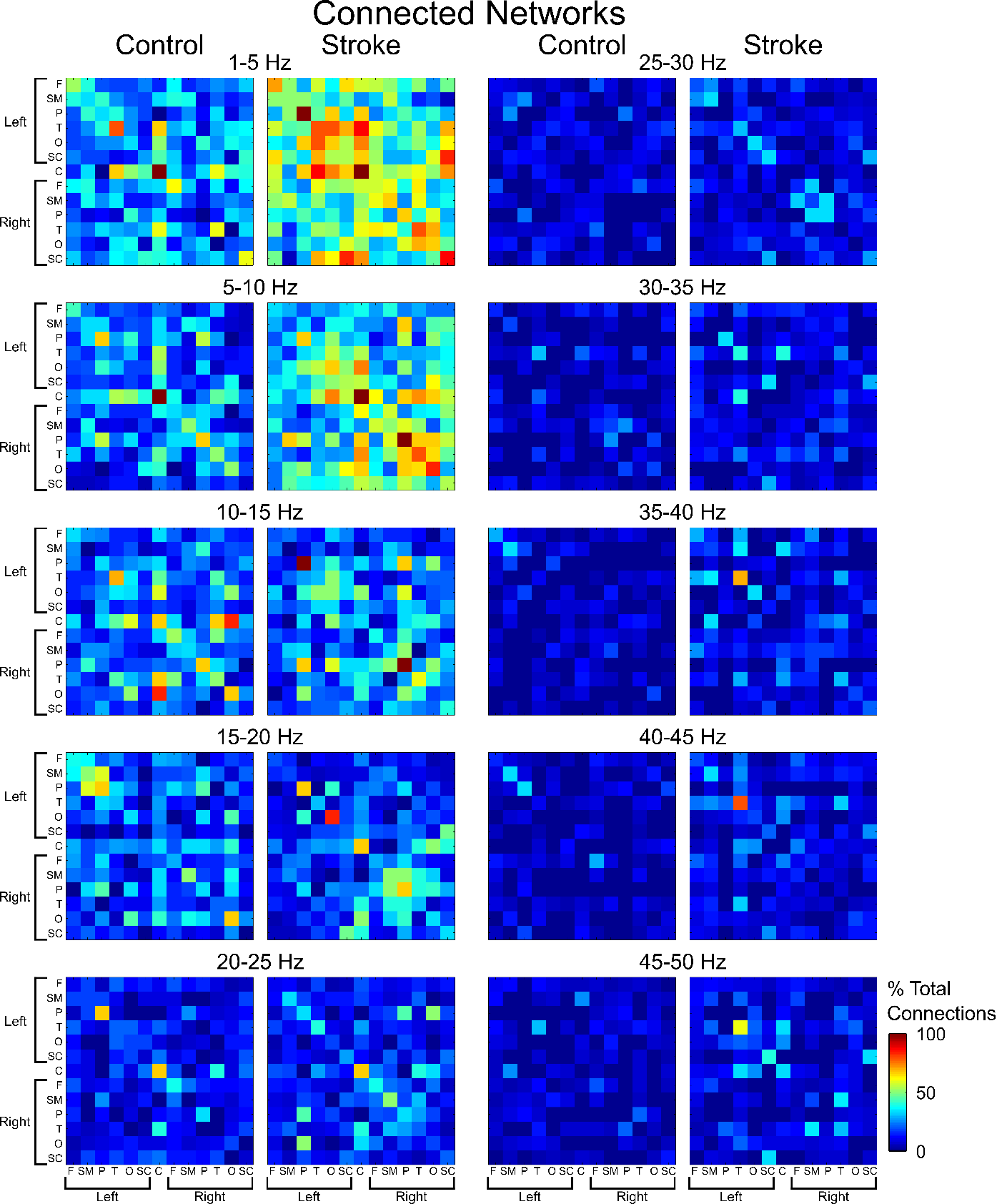


**Supplementary Figure 3:** Functional connectivity networks during resting state. The left hemisphere is associated with the stroke lesion. Networks deemed significantly connected within control (p<=0.0008) and stroke (p<=0.0002) groups (displayed in Figure 4) were grouped by cortical region (F: frontal, SM: sensorimotor, P: parietal, T: temporal, O: occipital, SC: sub cortical, C: cerebellum) for each frequency band of interest. The percentage of connections between and within cortical regions (based on the total number of possible connections within the cortical region or between the two cortical regions of interest) was then calculated and displayed.


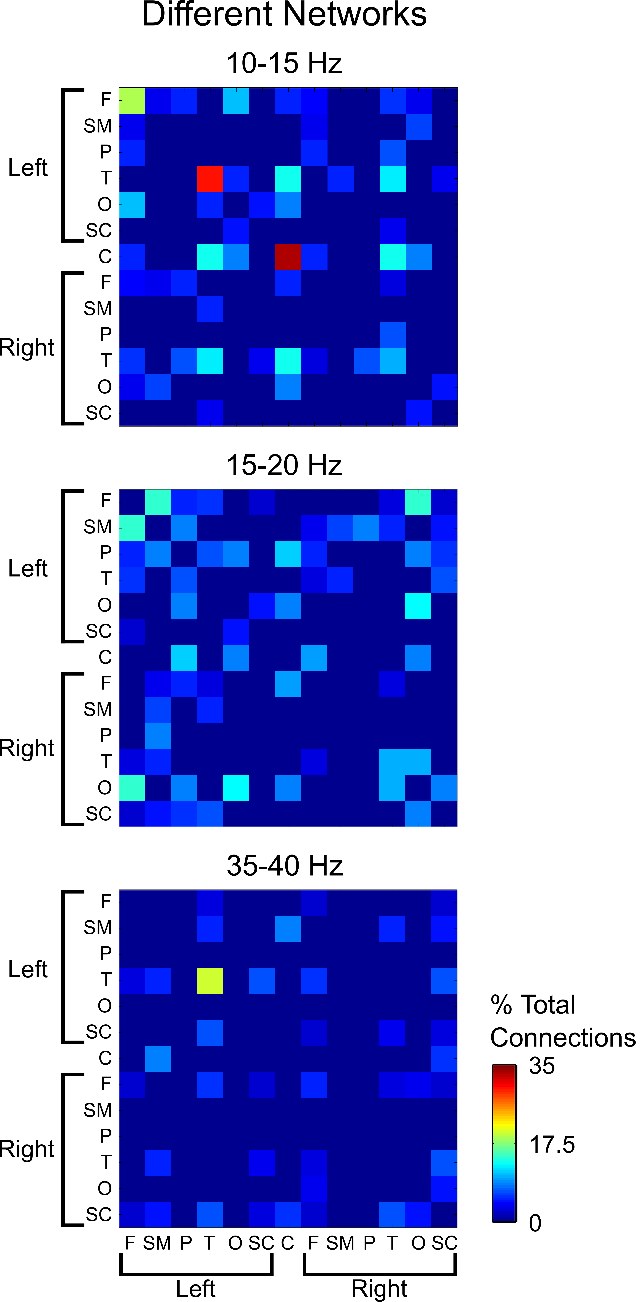


**Supplementary Figure 4:** Resting state functional connections with differences. The left hemisphere is associated with the stroke lesion. Networks with statistically significant differences (p<=0.05) between control and stoke groups (displayed in Figure 5) were grouped by cortical region (F: frontal, SM: sensorimotor, P: parietal, T: temporal, O: occipital, SC: sub cortical, C: cerebellum) for each frequency band of interest. The percentage of connections between and within cortical regions (based on the total number of possible connections within the cortical region or between the two cortical regions of interest) was then calculated and displayed.
